# Instrumental variable methods for a binary outcome were used to informatively address noncompliance in a randomized trial in surgery

**DOI:** 10.1016/j.jclinepi.2017.11.011

**Published:** 2018-04

**Authors:** Jonathan A. Cook, Graeme S. MacLennan, Tom Palmer, Noemi Lois, Richard Emsley

**Affiliations:** a Centre for Statistics in Medicine, Nuffield Department of Orthopaedics, Rheumatology and Musculoskeletal Sciences, University of Oxford, Botnar Research Centre, Nuffield Orthopaedic Centre, Windmill Road, Oxford, OX3 7LD, UK; b The Centre for Healthcare Randomised Trials (CHaRT), Health Sciences Building, University of Aberdeen, Foresterhill, Aberdeen, AB25 2ZD, UK; c Department of Mathematics and Statistics, Fylde College, Lancaster University, Lancaster, LA1 4YF, UK; d Wellcome-Wolfson Institute of Experimental Medicine, School of Medicine, Dentistry and Biomedical Sciences, Queens University, 97 Lisburn Road, Belfast, BT9 7BL, UK; e Centre for Biostatistics, School of Health Sciences, Manchester Academic Health Science Centre, The University of Manchester, Jean McFarlane Building, Oxford Road, Manchester, M139PL, UK

**Keywords:** Instrumental variable, RCT, Noncompliance, Binary, Causal modeling, Risk ratio

## Abstract

**Objectives:**

Randomization can be used as an instrumental variable (IV) to account for unmeasured confounding when seeking to assess the impact of noncompliance with treatment allocation in a randomized trial. We present and compare different methods to calculate the treatment effect on a binary outcome as a rate ratio in a randomized surgical trial.

**Study Design and Setting:**

The effectiveness of peeling versus not peeling the internal limiting membrane of the retina as part of the surgery for a full thickness macular hole. We compared the IV-based estimates (nonparametric causal bound and two-stage residual inclusion approach [2SRI]) with standard treatment effect measures (intention to treat, per protocol and treatment received [TR]). Compliance was defined in two ways (initial and up to the time point of interest). Poisson regression was used for the model-based approaches with robust standard errors to calculate the risk ratio (RR) with 95% confidence intervals.

**Results:**

Results were similar for 1-month macular hole status across methods. For 3- and 6-month macular hole status, nonparametric causal bounds provided a narrower range of uncertainty than other methods, though still had substantial imprecision. For 3-month macular hole status, the TR estimate was substantially different from the other point estimates.

**Conclusion:**

Nonparametric causal bound approaches are a useful addition to an IV estimation approach, which tend to have large levels of uncertainty. Methods which allow RRs to be calculated when addressing noncompliance in randomized trials exist and may be superior to standard estimates. Further research is needed to explore the properties of different IV methods in a broad range of randomized controlled trial scenarios.

**What is new?**

**Key findings**
• Across the different time points and noncompliance definitions for the case study, the point estimates of the various methods were generally similar.
• The nonparametric casual bound approach produced a narrow range of uncertainty than the risk ratio confidence interval of the two-stage residual inclusion instrumental variable (IV) method and standard intention to treat, per protocol and treatment received estimates.

**What this adds to what was known?**
• This article compared conventional analyses for dealing with noncompliance in a randomized controlled trial with two IV approaches for a binary outcome.
• The assumptions, advantages, and disadvantages of the approaches are considered using a surgical trial with substantial receipt of the nonallocated treatment over the follow-up period (“noncompliance”).

**What is the implication and what should change now?**
• The nonparametric causal bounds approach for binary outcome may be more informative than the standard method to address noncompliance in a trial in some situations.
• The generalizability of the finding should be explored across a range of settings and levels of treatment effect and noncompliance.

## 1 Introduction

Randomized controlled trials (RCTs) are widely seen as the optimal way to evaluate the effect of treatments. However, the design, conduct, and analysis of an RCT can undermine the purpose of randomization and introduce bias in the estimation of treatment effects. Departures from random allocation (often referred to as noncompliance or nonadherence) create uncertainty in the interpretation of findings with regard to the causal effect of treatment. Although an intention-to-treat (ITT)–based analysis remains the default analysis [1], [2], [3], in the presence of substantial noncompliance, it is natural to ask the question “what is the effect of actually receiving the treatment?”

Two common approaches used to address noncompliance are per-protocol (PP) and treatment-received (TR) analyses. Under a PP analysis, only data from those participants deemed to have complied with the (treatment) protocol are included. In a TR analysis, the analysis groups are formed on the basis of the actual TR, irrespective of the randomized treatment. The shortcomings of these conventional approaches to dealing with noncompliance are well recognized [1], [3]. Those who do not comply with treatment allocation (e.g. did not get surgery as allocated) tend to be different from the typical participant (they are often sicker and poorer in health, though in some situations the reverse can be true). A PP analysis excludes the subset of participants who do not comply from the analysis risk potentially introducing selection bias, as those who comply may reflect different patient characteristics between the groups. The TR analysis is carried out on the basis of transferring individuals who “crossed over” to the other group, and so also introduces bias into the comparison.

More recently, causal methods which address noncompliance while maintaining the integrity of randomization and avoiding exclusions of participants have been proposed [3], [4], [5], which vary in complexity and the underlying assumptions. Focus has mainly been on continuous outcomes [2], [4], [6], [7], [8] partly through the more ready application of methods, although approaches for binary outcomes do exist [1], [4], [5], [9], [10]. Their use has been limited, and when used, the focus has been on calculating the risk difference and in the setting of an observational study [11], [12]. In particular, causal bound instrumental variable (IV) methods have received little attention but can be readily calculated when the instrument, exposure variable, and the outcome are binary [9]. Surgery is considered an example of a scenario where compliance issues are “simple” (i.e., surgery is or is not received) as opposed to drug treatment or complex interventions which are delivered over time [5]. However, the recent work has highlighted the potential complexity of surgical interventions [13], [14], [15]. The use of compliance-based trial analyses in the area of surgery has been very limited to date, and methodological considerations have focused on surgery versus medicine and for a continuous outcome [16]. The aim of the work presented herein is to explore the compliance in a surgical randomized trial, where the treatment effect for a binary primary outcome is expressed as a risk ratio (RR). Through the case study, we seek to illustrate the use of randomization respecting compliance analyses versus conventional methods and to consider issues relating to compliance in this setting.

### 1.1 Case study–FILMS trial

The Full Thickness Macular Hole and Internal Limiting Membrane Peeling Study (FILMS) trial compared macular hole surgery with or without peeling (removal) of the internal limiting membrane (ILM) of the retina for idiopathic full thickness macular holes (FTMH) [17], [18]. Macular hole surgery, which seeks to close the hole and improve patient visual outcome, involves a number of steps with peeling an optional additional step. Patients with stage 2 or 3 FTMH were randomized to receive macular hole surgery with or without ILM peeling or not at nine centers. Of the 141 participants randomized, 138 were included in the statistical analysis (three were discovered not to meet the eligibility criteria after being randomized). The status of the macular hole (open or closed), the main surgical outcome, was assessed at 1, 3, and 6 months after surgery. Other outcomes collected included visual function (EDTRS visual acuity in the study eye [the primary outcome] and the fellow eye) and quality of life (Visual Function Questionnaire-25 and EuroQol 5 Dimensions 3 Levels). Principal (ITT based) study analyses found evidence of decreased occurrence of an open hole at 1 month but no statistical evidence of difference at 3 and 6 months [17]. However, interpretation of these findings was complicated by the occurrence of further surgery with 29 (43%) of the nonpeeling group received peeling within the 6-month follow-up period (Table 1). Some occurrences of peeling within this group were as per the initial treatment and some as a further surgical intervention, which was allowed in FILMS according to standard clinical care. Although a number of factors could have contributed to the loss of a significant effect at the latter time points (3 and 6 months), it was thought to be most likely caused by the substantial number receiving peeling in the control group. A later individual patient data meta-analysis which included the FILMS trial provided further support for this view [19]. Although participants receiving peeling in the no peel group if their initial treatment was deemed to fail can be viewed as both clinically necessary and allowable within the trial protocol, from an evaluation perspective, it can also be viewed as “noncompliance” and an obstacle to an accurate assessment of the effect of actually receiving the treatment.

**Table 1:** Number of participants who received peeling by randomized group (*N* [%])

Time from baseline	Peeling group (*n* = 71)	No peeling group (*n* = 67)
1 month	64 (90)	4 (6)
3 months	64 (90)	27 (40)
6 months	64 (90)	29 (43)

## 2 Methods

### 2.1 Scenario

**Fig. 1:**
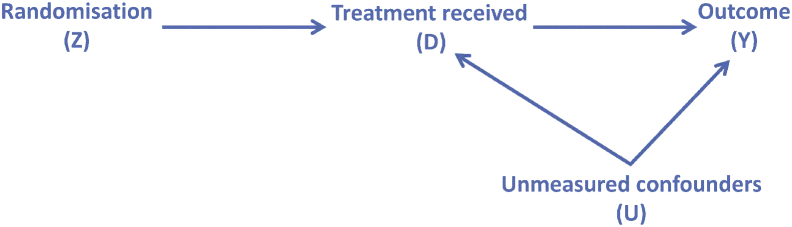
Graphical representation of a trial with noncompliance, with randomization as an instrumental variable for the treatment received.

The following considers the situation where we have a 2-arm RCT with treatment allocation (*Z*), a binary outcome (*Y*), and a binary measure of receipt of the treatment (*D*). We define compliance as a participant receiving their randomly allocated treatment (Z = D). In addition, we acknowledge the possibility of a vector of variables, *U*, which are unmeasured confounders of the outcome and compliance. Fig. 1 represents this graphically. An estimate of the treatment effect on outcome which accounts for the presence of noncompliance with treatment allocation is desired. The presence of *U* results in bias under conventional methods, such as excluding those who do not comply (PP) or direct adjustment in the statistical analysis. One way to deal with this is the use of an IV approach, in this setting of random allocation (*Z*), which enables the influence of *U* to be controlled for. A valid IV has to meet three standard IV assumptions: (1) the IV, *Z*, is assumed to be independent of *U*; (2) the TR (*D*) is assumed not to be independent of the IV (i.e., *Z*), which here implies that the random allocation influences the TR (*D*); and (3) the outcome (*Y*) is assumed to be independent of random allocation, given knowledge of the unobserved confounder and the TR, which implies that the random allocation only influences the outcome through the TR. This last assumption is referred to as the “exclusion restriction” [3]. A further assumption, most commonly a monotonicity assumption (e.g., the absence of “defiers”, individuals who will always receive the opposite of their random allocation), is assumed to enable the local average treatment effect, also called the complier average causal effect (CACE), to be estimated [20], [21]. Other distributional assumptions that can be made about the relationships of *Y* to *U* and *D* (e.g., additivity on the chosen outcome scale of interest and assuming a particular model form) are also commonly made to allow estimation of the point estimate and associated uncertainty [1]. For simplicity and to allow direct comparability between methods, the presence of observed confounders (such as might be used in the randomization algorithm, and in the case study, a prognostic factor such as stage of macular hole) has been ignored throughout, although the general approach can be extended, given appropriate assumptions to allow for controlling for these (e.g., through a regression model), as per the ITT analysis.

Compliance (and noncompliance) was defined as receipt (or not) of the allocated treatment, irrespective of the reason for any deviation from allocation. In our case study, three different definitions of compliance were used: (1) compliance to the presence or absence of peeling during the initial surgery (C1); (2) compliance to the undertake peeling or not up to 3 months after surgery (C2); and (3) compliance to the undertaking of peeling or not up to 6 months after surgery (C3).

### 2.2 Comparison of methods

The three classic conventional analyses were conducted; ITT, estimating the causal effect of random allocation where participants are analyzed according to the randomized groups irrespective of receipt of treatment (i.e., the effect of *Z* on *Y*); PP, where analysis was according to randomized group using the available data for those viewed as complaint (i.e., the effect of *Z* on *Y* in those for whom *Z* = *D*); and TR where the participants were grouped according to the actual TR up to the corresponding time point, irrespective of the random allocation (i.e., the effect of *D* on *Y*). It should be noted that the term “ITT” is not used here in the strict sense as there was a small amount of missing data for the primary outcome, and three ineligible participants were excluded postrandomization. For the purposes of this case study comparison, to match the trial's principal analysis, and for simplicity of presentation, the impact of this not been considered here. An unadjusted Poisson regression model with robust standard errors was used to calculate three conventional treatment effects and the associated uncertainty at the 5% 2-sided significance level (via 95% confidence interval [CI] and *P*-value) with the effect expressed as an RR.

In addition, two IV methods were used in which random allocation was used as an IV to allow an unbiased causal effect to be calculated in the presence of unobserved confounding factors (e.g., receipt of the treatment being influenced by another unmeasured factor such as surgeon treatment preference). The two IV analyses used were as follows:

1. A causal bounds (cbounds) approach in which bounds for the average treatment effect were calculated using the bpbounds command in Stata [9]. Under this approach, no point estimate is calculated but instead bounds for the causal effect for which a distribution exists given the underlying standard IV assumptions. Together, the IV assumptions impose constraints on the causal risk ratio for a binary outcome, and bounds can be calculated. In this context, it allows the bounding of the effect on receiving the treatment in the trial (“global”) population. Details on how this can be achieved are given in the Online appendix along with the additional invoking of a monotonicity assumption.
2. A two-stage residual inclusion estimator approach (2SRI) [1], [22]. This IV approach differs from the causal bounds approach in that further assumptions are made to estimate the point estimate of the causal effect. This leads to a local estimate of the treatment effect among those who would comply with the allocated treatments. Under this method, the residuals from a regression of TR on randomization (first stage) were obtained to enable an unbiased analysis of the effect of receiving treatment on outcome in the compliers (second stage). This second stage was carried out using an unadjusted Poisson regression model for the treatment effect by regression of the outcome on the TR (with 95% CI calculated) adjusted for the first-stage residuals as covariates. The model can be formulated as:

Y∼Poisson(r),

where $log_{e}\left(r\right)=\beta_{0}+\beta_{1}D+\beta_{2}E$ and *E* is the residual from the first-stage least squares linear regression of TR (*D*) to the instrument (*Z*) such that

$$E=D-\left({\hat{\alpha }}_{0}+{\hat{\alpha }}_{1}Z\right)$$

where ${\hat{\alpha }}_{0}$ and ${\hat{\alpha }}_{1}$ are the estimated regression coefficients from the first-stage regression.

The 2SRI estimate for a binary outcome is the same as the adjusted IV of Palmers et al. [10] and equivalent to Nagelkerke and colleagues' adjusted treatment received approach to estimate the causal effect in the presence of noncompliance [1]. After the second stage of estimation, the standard errors of the estimates were corrected using the method of Terza [23].

## 3 Results

### 3.1 Randomization as an IV in the FILMS study

Compliance status is presented in Table 1 using the aforementioned three definitions. Baseline values for key variables are presented by the randomized groups and/or by compliance status in Table 2. Macular hole closure findings (the outcome) are provided at 1, 3, and 6 months in Table 3. The proportion complying is similar at 1 month and markedly different at 3 and 6 months suggested that a PP analysis is at the risk of substantial bias when following the protocol is defined as through the follow-up period (C2 and C3). Regarding the IV assumptions, randomization, if correctly implemented, precludes the possibility of an association between random allocation and any pre-existing unmeasured confounders. For each of the compliance definitions, it is worth noting receipt of allocation to peeling was associated with greater receipt of peeling as would be anticipated in an RCT (i.e., more individuals allocated to peeling received peeling than those allocated to no peeling) supporting acceptance of core IV assumption (2). The distribution of the variables were checked to see if they were consistent with the core IV assumptions. Baseline data from the FILMS trial are presented in Table 2 by randomized group and by compliance status. Key trial outcomes did not appear to differ greatly by compliance, although this does not clarify the status of unmeasured cofounders which by definition cannot be directly checked. Given the two treatments of interest were the same bar, the additional step of peeling, the third IV assumption (3) would also seem very plausible.

**Table 2:** Full Thickness Macular Hole and Internal Limiting Membrane Peeling Study trial—baseline variables by randomized group and compliance groups at 6 months

Key baseline measures, mean (SD)	Randomized group		Compliance (6 months)		Compliers (6 months) by randomized groups	
	Peel (n = 71)	No Peel (n = 67)	Complier (n = 102)	Noncomplier (n = 36)	Peel (n = 64)	No peel (n = 38)
Age	70 (6)	71 (6)	71 (6)	71 (6)	70 (6)	71 (6)
Stage of hole (stage 3), *n* (%)	42 (59)	41 (61)	58 (57)	25 (67)	38 (59)	20 (54)
EDTRS visual acuity study eye	48 (14)	50 (11)	48 (10)	48 (10)	48 (14)	52 (11)
EDTRS visual acuity fellow eye	76 (14)	76 (17)	78 (11)	78 (11)	76 (14)	75 (20)
EuroQol 5 Dimensions 3 Levels	0.80 (0.21)	0.88 (0.13)	0.84 (0.14)	0.84 (0.14)	0.80 (0.21)	0.90 (0.13)
Visual Function Questionnaire-25	80 (16)	80 (18)	79 (21)	79 (21)	79 (16)	82 (13)

**Table 3:** Macular hole status by time point and randomized group (*N* [%] closed)

Time point	Randomized groups	
	Peeling group (*n* = 71)	No peeling group (*n* = 67)
1 month	56 (84)	31 (48)
3 months	61 (92)	52 (83)
6 months	61 (94)	56 (89)

### 3.2 Comparison of analyses

Table 4 shows the results using two compliance definitions for different methods for the various treatment effect measures. For macular hole status at 1 month, the ITT and TR, 2SRI and PP (under compliance to the initial surgery) analyses are all highly significant with large estimated RRs (between 0.26 and 0.32). The ITT effect is the smallest estimate. Interestingly, the causal bounds approach contains all the estimated point estimates (0.24, 0.38) but is narrower than all the estimated CIs. At 3 months, there is a similar pattern; although most of the analyses now show no statistical evidence of a difference. However, the causal bounds method shows evidence of a causal effect given initial compliance. This is no longer true if compliance is based on the absence of peeling through the 3-month follow-up period. For 6 months under initial compliance, all the regression analyses provide a similar point estimate likely due to no participants with 6-month data who both had an event and were noncompliant. The causal bounds method again shows weak evidence of a causal effect. Under compliance across the 6-month period, the 2SRI estimate is large than under ITT, PP, and TR. The causal bound approach no longer shows evidence of a causal effect. Across all 3- and 6-month analyses, the 2SRI estimates had a high degree of uncertainty larger than any of the other approaches.

**Table 4:** Risk ratio results (95% CI/causal bounds)

Outcome	Compliance	Method	Point estimate (lower–upper)	*P*-value
1 month		ITT	0.32 (0.18–0.58)	0.001
	C1	PP	0.26 (0.14–0.51)	<0.001
		TR	0.25 (0.13–0.49)	<0.001
		2SRI	0.26 (0.13–0.53)	<0.001
		Causal bounds	NA (0.24–0.38)	
3 month		ITT	0.43 (0.16–1.18)	0.103
	C1	PP	0.35 (0.12–1.05)	0.061
		TR	0.33 (0.11–0.97)	0.044
		2SRI	0.37 (0.11–1.26)	0.112
		Causal bounds	NA (0.27–0.69)	
	C2	PP	0.35 (0.11–1.12)	0.078
		TR	0.48 (0.19–1.20)	0.117
		2SRI	0.20 (0.02–1.60)	0.129
		Causal bounds	NA (0.13–1.09)	
6 month		ITT	0.55 (0.17–1.81)	0.328
	C1	PP	0.55 (0.17–1.80)	0.326
		TR	0.55 (0.17–1.81)	0.328
		2SRI	0.51 (0.13–1.99)	0.331
		Causal bounds	NA (0.39–0.97)	
	C3	PP	0.45 (0.13–1.68)	0.214
		TR	0.51 (0.16–1.66)	0.238
		2SRI	0.31 (0.03–3.84)	0.362
		Causal bounds	NA (0.12–1.36)	

*Abbreviations:* CI, confidence interval; ITT, intention to treat; PP, per protocol; TR, treatment received; 2SRI, two-stage residual inclusion approach.

## 4 Discussion

Departures from random allocation can create uncertainty about the interpretation of trial results based on an ITT analysis. Various approaches have been proposed to allow an estimate of the effect of TR. We illustrated the use of four approaches for a binary outcome based on estimating an RR, including two IV-based methods. Binary outcomes are often one of, if not the key outcome in randomized trials [24], and compliance to treatment allocation can rarely be taken for granted, which suggests that the potential use of such methods (including the causal bounds approach) as an ancillary analysis potentially have widespread application. A similar bounding approach can in principle be applied to a continuous outcome where a minimum and maximum value can be assumed for the outcome, and further, more restrictive assumptions were made [25]. The two-stage least squares IV approach can also be used for a binary outcome to estimate the risk difference although it does not constraint the probability between zero and one [6]. The IV approaches used here can and have been used in Mendelian randomization epidemiology studies [9], [10].

Interestingly, in our analyses, the causal bounds IV approach produced a more precise and potentially useful finding than the other IV-based analysis (2SRI), which had very wide CIs. In addition, it also produced a more precise estimate of the causal effect than the TR and PP analyses as well as requiring less strong assumptions regarding those who comply with allocation (i.e., absence of selection bias). Two-stage residual inclusion approach makes an additional assumption of linearity on the scale of interest which may not be appropriate [1], [10]. Similarly, a two-stage fully linear approach could have been adopted although it has been demonstrated this may not produce a consistent estimator for ratios but could have been used to calculate the risk difference [6], [26]. Appropriate CIs are not straightforward for any of the model-based IV analyses further providing merit to the nonparametric causal bounds approach. The lack of a point estimate could be viewed as a substantial disadvantage of a causal bounds approach. Accordingly, such approaches are sometimes referred to as “partial identification” methods [25]. However, it is worth noting the level of uncertainty around the point estimate from some of model-based IV analyses can reduce the corresponding point estimate to little practical value.

The disadvantages of the PP and TR analyses are well known [5], and the assumptions required to provide an unbiased analysis are often implausible (those who comply do not differ in their characteristics from those who do not comply). IV approaches avoid making this assumption while preserving the benefits of randomization. However, they require additional assumptions beyond that of randomized treatment allocation. The plausibility of these assumptions will vary according to the situation. In the FILMS trial example, the assumption that randomization influence the TR is not problematic. Similarly, the exclusion restriction assumption also seems very plausible given both treatment arms receive a surgical operation which differs only in respect to a single component (peeling or not of the ILM). A minor caveat is the small number of patients did not have any surgery or additionally received another surgical intervention. The remaining IV assumption regarding independence of the unmeasured confounders and the IV, is partially met at least by random allocation, but, it is possible that allocation had some impact on such factors particularly given it was not possible due to the nature of the trial to fully blind all individuals involved in the study. The adjusted treatment received method required further assumptions of monotonicity, and in our implementation, additivity of effect of TR and the unmeasured confounders. In this example, the benefits of the simple application of the 2SRI approach over a causal bounds approach is dubious for the simple binary outcome setting. This problem of a lack of precision applies more generally to approaches which estimate the CACE [5], [11]. The large effect of macular hole surgery (with or without) peeling is perhaps atypical though somewhat offsetting this was the relatively small size of the trial. Recent work has focused on attempts to increase the precision of such analyses given the large level of uncertainty reflected in our analyses [5].

The change or not in outcome status over time as treatment is received could provide a more precise estimate of the treatment effect. A recent study compared a number of methods (including IV and mixed model approaches) for addressing noncompliance of a medical vs. surgical trial compared in the setting of a continuous outcome with repeated measures [16]. In principle, a similar approach could be carried out for a binary outcome which makes use of the repeated data although the robustness of any formal interference (i.e., calculating a 95% CI) is unclear. Another aspect not addressed here is the complication generated by the presence of missing data. This could affect both the precision of the estimates and also introduce bias. Further research on this topic in needed to explore this.

Surgery might be considered a simple situation to address compliance as it can be considered a one-off event, which either occurs or does not [5]. However, even in this context, we showed that different definitions of compliance are possible. The reality of surgical trials, similar to many other skill-dependent interventions in particular, does not fully fit this situation as the intervention can sometimes be repeated or delivered over a number of occasions, and varying ways. The analyses reported here in a less complex surgical trial (surgery versus surgery which differs only in a minor way [27]) still make the simplification that peeling of ILM was or was not carried out. However, a small number of participants did not receive any surgery or received another surgical intervention, or received another nontrial surgical procedure during the follow-up period. In addition, it is possible to repeat ILM peeling if the initial peeling was not complete as occurred in two cases; similarly, this was also not accounted for in the analysis. Finally, while we have focused implicitly on more surgery orientated compliance, the full intervention included a patient-dependent postoperative component of posturing (lying face down after the operation for prolonged periods of time) [17], [28]. Limitations in data collection and reporting hinder more in-depth assessment of the impact of this aspect of compliance. Nevertheless, the methods here provide at least some statistical reassurance that the presumed interpretation of a casual treatment effect of ILM peeling is correct even though the ITT analyses did not always reflect this.

In conclusion, this study has illustrated the use of different IV methods, which can be readily used to supplement the ITT analysis to address compliance for a binary outcome with the treatment effect expressed as an RR. A surgical randomized trial was used to illustrate their use, and the nonparametric causal bounds approach provided more certainty about the treatment effect once accounting for noncompliance. However, as highlighted, these methods require further assumptions to be made beyond the ITT analysis to assess a causal effect of treatment, and they tend to produce imprecise estimates despite representing a simplified scenario of the reality of compliance in a surgical setting. Nevertheless, their use can aid the interpretation of surgical trials in the presence of noncompliance, and we encourage the more routine adoption of these techniques, which allow the impact of compliance to be evaluated without compromising the benefits of randomization.
