# Supplementary material for: Instrumental variable methods for a binary outcome were used to informatively address noncompliance in a randomized trial in surgery
Source: J Clin Epidemiol. 2018 Apr;96:126–32. doi: 10.1016/j.jclinepi.2017.11.011 (PMC5862096; doi:10.1016/j.jclinepi.2017.11.011)
Supplement: Online Appendix [file mmc1.docx]

**Online appendix**

Constraints can be placed upon the causal bounds given the three standard instrumental variable assumptions Ai)-Aiii) which are stated in the main body of the paper. The probabilities of a successful outcome for Y can be defined (given treatment received *D*) as:

$$\pi_{d}=P\left\{ Y=1 | D=d \right\}$$

and the conditional probabilities are defined as:

$$p_{yd.z} P\left( Y=y, D=d | Z=z \right)$$

The causal risk ratio (CRR) can be shown to be:

$$\frac{\pi_{1}^{L}}{\pi_{0}^{U}}\leq CRR\leq\frac{\pi_{1}^{U}}{\pi_{0}^{L}}$$

where $\pi_{d}^{L}$ and $\pi_{d}^{U}$ the lower and upper bounds respectively of the corresponding statement for $\pi_{d}$:

$$max\binom{{p_{10.1} \atop p_{10.0}}}{{p_{10.0}+p_{11.0}-p_{00.1}-p_{11..1} \atop p_{01.0}+p_{10.0}-p_{10.1}-p_{11.1}}}\leq\pi_{0}\leq min\binom{{{1- p}_{00.1} \atop{1-p}_{00.0}}}{{p_{01..0}+p_{10..0}+p_{10..1}+p_{11..1} \atop p_{10..0}+p_{11.0}+p_{01.1}+p_{10.1}}}$$

And

$$max\binom{{p_{11.0} \atop p_{11.1}}}{{{-p}_{00.0}-p_{01.0}+p_{00.1}+p_{11..1} \atop{-p}_{01.0}+p_{10.0}+p_{10.1}+p_{11.1}}}\leq\pi_{1}\leq min\binom{{{1- p}_{01.1} \atop{1-p}_{01.0}}}{{p_{00..0}+p_{11..0}+p_{10..1}+p_{11..1} \atop p_{10..0}+p_{11.0}+p_{00.1}+p_{11.1}}}$$

The bounds can potentially be tightened by making a further assumption of monotonicity such that for all values *u* of *U*

$$P\left( D=1 | Z=1,U=u \right)\geq P\left( D=1 | Z=0,U=u \right)$$
